# Tackling upcoming projects: The development and efficacy of event previews an experimental study

**DOI:** 10.1371/journal.pone.0293271

**Published:** 2023-12-18

**Authors:** Eveline Schollaert, Shana Mertens, Frederik Anseel, Tom Kluijtmans, Marie Servaes, Saskia Crucke

**Affiliations:** 1 Department of Marketing, Innovation and Organization, Faculty of Economics and Business Administration, Ghent University, Ghent, Belgium; 2 UNSW Business School, University of New South Wales, Sydney, Australia; National University of Sciences and Technology, PAKISTAN

## Abstract

Traditional performance management systems are increasingly seen as ill-conceived for today’s dynamic organizational landscape. Researchers and practitioners advocate for agile PM systems that emphasize continuous monitoring, learning, and feedback. We present the ‘event preview’, a novel approach that is designed to address several shortcomings of traditional performance management practices. Event previews consist of five fixed questions, which are discussed among team members before an event, instigating a detailed reflection and mental simulation of upcoming events or projects in order to achieve the desired outcomes. In doing so, event previews support teams to utilize their projects as learning opportunities. This study provides the theoretical basis for the event preview and empirically tests its effectiveness. A sample of 119 teams participated in the experiment in which they were asked to solve as many puzzles as possible within a fixed time frame. One condition conducted an event preview beforehand, the other condition did not. Our findings, which were based on a comparison of the averages of the two conditions, suggest that the event preview holds promise for improving team performance and communication. As such, the event preview presents an additional instrument to the changing performance management landscape. This simple practice can be incorporated in the performance management cycle, emphasizing adaptability and continuous improvement in organizations.

## Introduction

Performance management (PM) has been under pressure for several years. Researchers argue for a reevaluation of its relevance in today’s organizational landscape. According to some scholars, the traditional formal PM system–and especially the periodic evaluation interviews or appraisals that are central to it—no longer accommodate the day-to-day operations of organizations and is ill-suited for accurately gauging performance [1, 2]. The traditional employee feedback and appraisal approach has its origin in a time where organizations and the business environment were characterized by organizational stability, hierarchical structures, and a centralized control [3]. In other words, traditional PM systems have not kept up with the dynamics of contemporary workplaces. Nowadays, for instance, many organizations apply a project-based approach instead of a yearly cycle [1, 4]. This requires them to incorporate fast and immediate feedback, rather than relying on an annual progress review [5]. Consequently, there is a clear need for innovation within this subject. Scholars and practitioners advocate for the creation of more agile PM systems. Central to these types of systems are the principles of continuous monitoring, frequent and iterative feedback processes, and an emphasis on continuous learning [6, 7].

Bearing this in mind, a set of inventive PM practices have been introduced to improve performance and drive employees’ professional development. One of these practices is the after-action review (AAR): a systematic review of the team’s performance during recently completed tasks or events [8]. The AAR has been shown to be effective; experimental studies suggest that conducting an AAR positively affects team performance, team efficacy, openness of communication, and other outcomes [9]. However, criticisms about the usefulness of the AAR in non-recurring projects, as well as its past-focused approach, create the need to introduce new tools and practices to facilitate project monitoring, feedback, and constant learning.

Therefore, by combining developmental principles of the AAR with psychological theories of mental contrasting by Oettingen [10], and construal level theory (CLT), we aimed to develop a new forward-looking approach: the event preview. When conducting an event preview, individuals in a team reflect systematically on how they will tackle an event or activity that will take place in the near future in order to achieve the desired outcomes. Objectives are set after cooperatively constructing a mental simulation of the event or project. Consequently, factors that may facilitate the desired results are identified and used to develop strategies. The event preview thus extends traditional goal setting approaches by constructing a mental image of how the event could unfold in the future, reflecting on the desired results, and creating a plan of action based on this mental simulation of the future. Consequently, the event preview answers several of the previously mentioned criticisms and can be presented as a practice that may facilitate (team) learning and monitoring of projects.

In this study, we clarify the theoretically based rationale for the development of the event preview practice and detail the different aspects. Additionally, we aim to test the effectiveness of our novel approach in a team cooperation context and investigate whether conducting an event preview can indeed improve teams’ performance. To do this, the event preview was tested using an experimental design testing its relationship with three team-related outcomes, namely task performance, openness of communication, and team efficacy. In this study, a sample of 119 three-person teams participated in an experiment in which they tried to solve a task within a fixed period of time.

## Theoretical background

As mentioned, traditional performance management (PM) are under scrutiny. PM is the process of measuring, communicating, and managing employee performance to align performance with the organizational strategy [11]. The PM cycle consists of aspects such as planning, monitoring, and measuring performance, evaluating, and recognition or reward. The first step in the cycle would be planning: this includes goal setting and determining objectives for the upcoming period, both with regard to the development of certain skills and the achievement of specific quantitative (e.g., sales) results. It also encompasses planning how to reach these goals. These plans and objectives are monitored throughout the process. Finally, progress is measures, evaluated, and–if applicable and relevant–rewarded. Traditionally, the PM process was centered around periodical (often annual) progress reviews. In these conversations–that often implied a formal form to be completed, contributing to its bureaucratic nature–last year’s performances are evaluated and discussed, and new objectives are set and planned [6, 12]. Recently, however, scholars have emphasized the importance of moving toward adapted PM systems in which appraisal and performance evaluation are seen as ongoing processes rather than once-a-year events [13]. That is, nowadays organizations operate in a volatile environment where continuous change, adaptations, and learning are imperative for employees. Therefore, PM systems should focus on goal setting, cooperative learning, and continuous monitoring or feedback to create a developmental human resource management system [4, 6, 7].

In light of these challenges, one innovative PM practice that has been proposed to encourage ongoing dialogue and learning is the after-action review (AAR). The AAR, originating from the military, is a practice in PM that can be conducted after a task to facilitate feedback and reflection. Researchers found that conducting an AAR had positive effects on several team-related outcomes, such as performance and openness of communication [8, 9, 14]. This makes the AAR a particularly interesting practice for similar projects or events that recur regularly, as individuals learn from previous events and use this knowledge in similar situations. Although it is argued that new PM practices should be aimed at upcoming and changeable events [1, 15], the AAR is mainly past-focused, as it is performed after the event has occurred and is aimed at reflecting on prior performance. Moreover, in practice, teams often have to deal with important uncertain events that may occur only once. For these non-recurring projects, an AAR is of lesser value, as it more focused on reflecting on prior errors instead of focusing on how to anticipate them in the future. Thus, there is a clear need for a practice that is forward-looking and can also be helpful in unrepeated events.

This is where we suggest event previews have a role to play. An event preview considers the need for constant learning and continuous dialogue in today’s organizations, while also focusing on the future. Therefore, we argue that the event preview can be an addition to traditional PM practices (among which the AAR) as it may facilitate continuous learning, monitoring, and feedback, which has been previously advocated [1, 13]. In the following sections, the underlying theories that helped shape event previews will be discussed, this includes CLT and the strategy of mental contrasting by Oettingen [10]. These theories clarify the rationale behind event previews and the recommendation to make PM practices more forward-looking.

### Theoretically based rationale for the development and effectiveness of event previews

Many PM practices in organizations are based on monitoring performances and providing feedback. As the term itself indicates, although feed’back’ can be interesting to facilitate learning, it is still aimed at ‘looking back’ to reflect on what has already occurred, rather than focusing on the many possibilities for the future [16]. Additionally, when feedback includes negative information, people can feel threatened and respond defensively [17]. Generally, when people receive feedback with regard to past performances, there is an interaction between two categories of self-evaluative motives: self-protection and self-change [17]. This is a paradox, as these motives can be in conflict if a person is exposed to negative information about themselves [18]. Self-protection aims to preserve a positive image and confirmation of the self-concept, while self-change aims to improve the self-concept using information about one’s strengths and weaknesses [19]. This may explain the defensive reactions that sometimes occur when receiving negative feedback, as the need to think positively about oneself, and thus self-protection, is one of the strongest motives [17–19].

Recently, researchers have linked construal level theory (CLT) and the theory of psychological distance to the two categories of self-evaluative motives [17, 20]. Whereas high-level construal (i.e., thinking abstractly, looking at the bigger picture) is associated with the acceptance of feedback to facilitate self-change and learning, low-level construal (i.e., thinking more concretely, detailed) is associated with the dismissal of this feedback to serve self-protection [17]. In other words, people might better engage in high-level construal to accept feedback and to be able to learn. In this case, individuals manage to take a certain psychological distance from the present stressful demands and concentrate on their goals and the encompassing implications of their behavior [17, 20]. Additionally, researchers found that high-level construal can indeed encourage the acceptance of feedback–and can thus foster learning -, provided that the feedback is relevant and addresses changeable behavior [17]. Building on construal level theory, we argue that feedback that is aimed at the future will be more easily accepted because it increases psychological distance and is thus ‘safer’ for self-evaluation than feedback about past behavior.

Researchers suggest shifting the focus from the past to the future by incorporating mental simulations of the future and performance targets [15, 21]. A practice that responds to the ‘past-focused’ criticism is the feedforward interview (FFI), an interview in which the employee is asked to reflect on a positive experience in the past [16, 22]. By discussing this positive experience, one can identify the circumstances that facilitated the successes and use this information for future situations and challenges. The FFI is thus strongly oriented toward the future by focusing on the positive aspects of a situation, and how to repeat them in the future, instead of fixating on past errors [22]. An event preview presents a similar conversation; by talking through the future event, the circumstances that will likely facilitate success are identified. This helps individuals construct a mental image of the event, formulate the desired results, and develop possible strategies to achieve them. However, while the FFI is a very broad conversation between an individual and a supervisor concerning one’s career and positive experiences, an event preview is a structured collective reflection on a specific upcoming event or project. It is conducted in a team of collaborating individuals who may or may not be familiar with each other (e.g., new or existing teams), with a supervisor present or not. Furthermore, unlike the FFI, an event preview not only covers favorable circumstances but also reflects on the circumstances that could contribute negatively to the achievement of the desired results. Teams conducting an event preview thus construct a comprehensive mental simulation of the upcoming event.

Moreover, the event preview differs from the FFI as it involves formulating a concrete mental simulation, objectives, and strategies. This aspect of the event preview can be substantiated by the strategy of mental contrasting [10, 23, 24]. The authors state that mentally contrasting reality and a desired future will determine whether an individual will actually pursue a certain objective [25]. When there is a contrast between reality and the image of a desired future, the individual assesses the expectations of success. Based on this assessment of success, one will pursue the desired future [10, 24]. This expectancy-based route facilitates the necessity to act [24, 26]. The necessity to act only occurs when the discrepant cognitive elements of the desired future and current reality are noticed and explicitly elaborated [24].

Someone that holds expectations about a desired future, and thus experiences this discrepancy, can also be understood as someone who is confronted with a problem [24, 27]. A clear image of the desired future needs to be developed in order to achieve the desired outcomes and ‘solve’ this problem. To achieve the desired outcomes, one must hold expectations about a desired future and experience a necessity to act, as favorable expectations cause strong objective commitment [24]. The event preview practice uses these insights, as constructing a mental simulation of the future event and reflecting on the desired outcomes are crucial in this practice. We assume that, when we apply mental contrasting to multiple individuals, such as groups or teams, the process of discussing these mental contrasts and desired futures may be beneficial. Namely, the conversation can ensure that the possible scenarios or pitfalls of a project or event are uncovered and elaborated.

### Defining the event preview

**Fig 1:**
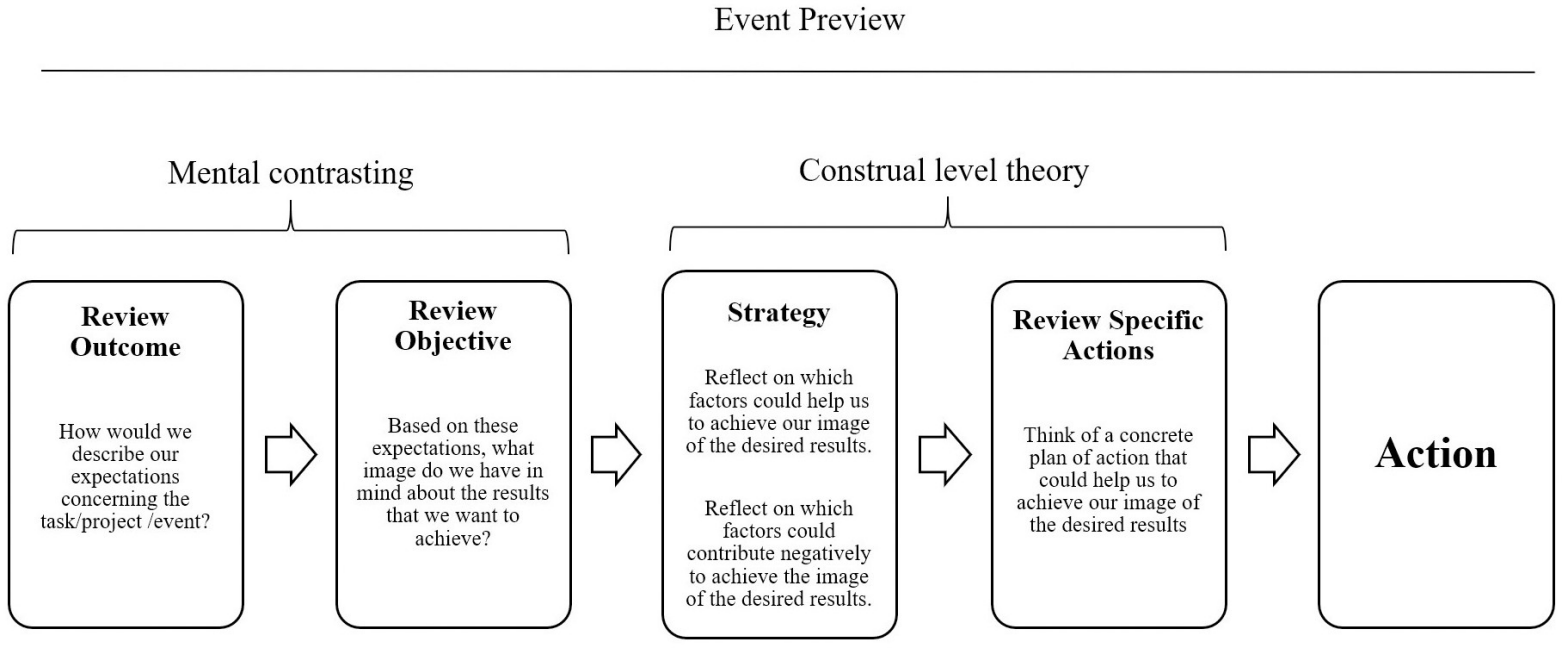
The components of the event preview.

Based on CLT, self-evaluative motives, and strategies of mental contrasting, the event preview can be defined as a structured way to collectively reflect on a future event or project, in order to successfully achieve the desired output. With this, an event preview encourages learning within the team. Ideally, event previews are conducted regularly, in order to create a learning culture in the organization [28, 29]. Similar to the AAR, and as illustrated in Fig 1, the event preview consists of a fixed set of five questions that are discussed by the team regarding the project or event. The first two questions asked during the first and second phases of the event preview are (a) “How would we describe our expectations concerning the task/project/event?” and (b), “Based on these expectations, what image do we have in mind about the results that we want to achieve?”. These questions were formulated according to the principles of the strategy of mental contrasting [10]. In this phase, team members construct a mental image together, bearing in mind the desired results of the event or project [24]. As mentioned, this may consequently increase performance [26].

During the third and fourth stages of the event preview, team members can (from a ‘safe’ psychological distance) reflect on this mental simulation and the possible implications of their behavior. Due to the future-looking nature of the conversation [30], individuals engage in high-level construal [17, 20]. During these stages, which are evidently related to CLT, three further aspects are discussed: (c) “Reflect on which factors could help us to achieve our image of the desired results”; (d) “Reflect on which factors could contribute negatively to achieve the image of the desired results”; and (e) “Think of a concrete plan of action that could help us to achieve our image of the desired results.” Event previews include a structured discussion in which team members reflect on the upcoming event to create a mental simulation, define their objectives, and explore a concrete plan of action.

With this, an event preview goes beyond the practice and theory of goal setting [31]. When conducting an event preview, the idea of mental simulation is central. The outcomes of the event are still unclear, and it is thus necessary to construct a mental image of the future event or project. Consequently, the expectations and desired results are determined based on this mental simulation. Additionally, the concrete actions and factors that may facilitate this desired future are uncovered, and strategies are developed.

The idea of considering the different possible outcomes of an upcoming event has been introduced previously. Klein [32], for example, introduced the concept of the PreMortem. When conducting a PreMortem, team members are asked to assume that the project has failed spectacularly and try to generate plausible reasons for the project’s failure [32]. The goal of this approach is to identify potential mistakes or obstacles beforehand, in order to prevent them during the project. Therefore, using this method ensures the identification of quality changes in the plan [33, 34]. However, the PreMortem has a focus on failure (and avoidance of failure), which contrasts with that of an event preview which is more success oriented. For instance, when individuals or teams conduct an event preview, they do not solely expose the risks but also determine strategies for success. By doing this, the main focus is on the mental simulation of possible positive outcomes and how these can be achieved. In other words, the goal of an event preview is to identify obstacles and success factors in order to construct a mental simulation and determine an appropriate strategy, rather than simply preventing potential mistakes. Thus, while identifying obstacles is pertinent in both approaches, the goal of a PreMortem is to overcome these obstacles, while the goal of the event preview is to use these insights as a means to build and select a strategy. Moreover, when conducting an event preview, team members define the desired future and ensure that the others are on the same page. While the PreMortem mainly functions as a cognitive analysis of future events and uncovers what should be avoided, the event preview approaches the desired future and may even have an energizing effect.

Additionally, Klein [32] states that the PreMortem is conducted by all team members separately, after which the team leader processes their ideas. He reports that: “those in the room independently write down every reason they can think of for the failure. […] After the session is over, the project manager reviews the list, looking for ways to strengthen the plan.” (p. 18). This is another considerable difference between both practices as the event preview is conducted by team members that cooperatively create a mental simulation of the event. Team members explicitly reflect and deliberate together to construct a mental image of the desired results. In other words, the interaction between the team members is a crucial aspect of event previews. The event preview thus partially builds on the insights of the PreMortem, but it extends the focus and main purpose of the practice. Therefore, this study intends to examine the value of an event preview and its effect on multiple outcomes.

### Hypotheses development

The main objective of this study is to experimentally compare the effectiveness of conducting and not conducting an event preview in a team before a task or project. We suggest that the mental simulation of future events, as performed in an event preview, will have additional positive effects. We thus suspect the event preview to be effective in improving teams’ task performance, through reflecting on desired outcomes and safely discussing possible actions. In addition, conducting an event preview among team members may affect other relevant team-related outcomes. It is expected that the event preview, a structured deliberation, will affect the perceived degree of a team’s openness of communication. It is likely that, in an organizational context, this will encourage open dialogue, which can also contribute to creating a learning culture.

Furthermore, clearly defining the desired future as a team and having a better understanding of the common objectives have been suggested to affect team members’ perceived team efficacy [35]. Sawyer [36] built on role theory and found that objective clarity and process clarity can cause team members to communicate more effectively with each other. This, in turn, could positively affect their perceived team efficacy [36, 37]. In other words, we suspect that the openness of communication can in fact mediate a positive relationship between the event preview condition and level of team efficacy.

Building on the preceding empirical literature and theories, we propose the following hypotheses:

*Hypothesis 1*: Compared with non-event preview teams, event preview teams will have higher task performance.
*Hypothesis 2*: Compared with non-event preview teams, event preview teams will report higher levels of openness of communication.
*Hypothesis 3*: Conducting an event preview will positively influence team efficacy, and this relationship will be mediated by the level of openness of communication.

## Method

### Participants

The participants could voluntarily register for this study. In the month before the experiment, potential participants were informed of the experiment using a snowball method and public Facebook posts. Participants were able to sign up as a team of three by sending a message or email. We specifically opted for three-person teams in order to homogenize the team size. This allowed the teams to have equal opportunities of achieving high scores throughout the game, especially since the relationship between team size and performance has been debated [38, 39]. Participants were randomly assigned a team number, which was used as a unique identifier in the surveys; this way, responses of team members could be linked to each other.

Participants received all necessary documents and completed the experiment as a team. Afterwards, they were asked to provide their answer sheet to the researchers, as well as fill out the digital survey (one survey per team member). The first page of the digital survey contained a consent form with all relevant information with regard to the experiment. Although nothing was disclosed about the different conditions, participants were briefed that they participated in a study on teamwork and team efficiency. It was clearly stated that, by continuing the survey, participants agreed that their responses would be used as described in the consent form. Furthermore, participants were informed that they were allowed to stop the experiment at any time, that their participation and responses were entirely confidential, and who to contact if they were to have any questions about the study.

The total sample consisted of 357 individuals who participated in 119 three-person teams. More teams volunteered but these teams either dropped out throughout the experiment or did not complete the surveys. Of the final sample, 40.1% were men and 59.9% were women. The average age of participants was 29.10 years (SD = 12.08 years). The overall and condition-specific demographic information is shown in Table 1.

**Table 1:** Demographic composition of the sample by condition.

Variable	Non-event preview		Event preview		Overall	
	*n*	*%*	*n*	*%*	*n*	*%*
Sex						
Female	112	59.3	102	60.7	214	59.9
Male	77	40.7	66	39.3	143	40.1
	*M*	*SD*	*M*	*SD*	*M*	*SD*
Age (in years)	29.36	12.62	28.82	11.47	29.10	12.08

*Note*: N = 357.

### Procedure

For this experiment, a game was developed in which participants had to solve a fictional murder. To solve the murder, participants had to work as a team to find answers to 20 riddles and puzzles. The solution for each puzzle provided them with the answer to a major question to solve the murder, for example: “What was the murder weapon?” or “What was the killer’s motive?” The puzzles contained rebuses, mathematical puzzles, (cross)word puzzles, sudokus, and logic puzzles. They ranged from relatively easy to relatively difficult. When participants volunteered to participate in the study, they were assigned a team number and received the necessary documents to complete the experiment. Team numbers were assigned to later match the performance scores to the survey answers. The documents each team received included clear guidelines to accomplish the task, together with a clear indication of the time limits, the puzzles, a response form, and a link to the online survey.

An experimental between-subjects design with two conditions was used to empirically test the effect of the event preview. Teams were randomly assigned to one of two conditions: teams that did not conduct an event preview and teams that did conduct an event preview. The latter were clearly instructed to discuss five questions during a ‘team deliberation’ of minimum five and maximum seven minutes. After the event preview, they were also asked to ‘solve the murder’ and fill in the response form and survey. To check the manipulation, teams in the event preview condition were requested to submit the concise notes of their event preview in addition to their response form. These notes were reviewed to ensure that these teams conducted the event preview properly. When notes were not submitted or were too limited, teams were excluded from analyses, as it was impossible to determine whether the event preview was conducted properly. Based on this evaluation, three of the initial teams were excluded, so that the 119 remaining three-person teams formed the total sample.

### Measures

#### Task performance

Performance scores were obtained based on the response forms. Researchers manually reviewed these documents and assigned a task performance score to each group. This score was simply based on the amount of correctly answered riddles. For example, a team that answered 16 questions, of which 12 were correct, were assigned a performance score of 12.

#### Openness of communication

Openness of communication was assessed using the four-item scale of Barry and Stewart [40], as previously used by Villado and Arthur [9] in their AAR study. Their group process measure was modified to suit our study and performance task. A sample item is: “Members are free to make positive/negative comments.” Each item is rated on a 5-point scale. The internal consistency estimate for the scale was .63.

#### Team efficacy

In this study, team efficacy was measured using a modified version of the 3-item measure used by Arthur, Bell [41], as previously used by Villado and Arthur [9] in their AAR study. A sample item is: “How confident are you in the ability of your team to solve the puzzles?” Items were rated on a 5-point scale. The internal consistency of the measure was 69.

## Results

All individual-level variables, namely, openness of communication and team efficacy, were aggregated to the team level. Agreement and reliability indices suggested that aggregation at the team level for both openness of communication and team efficacy was appropriate; *r*_*wg*_,ICC_1_ and ICC_2_ values for openness of communication were 0.91, 0.32, and 0.58, and for team efficacy were 0.87, 0.44, and 0.70, respectively. Task performance scores were assigned at the team level. The team-level means, standard deviations, and correlations for all variables are shown in Table 2.

**Table 2:** Team-level variable means, standard deviations and correlations.

Variable	*M*	*SD*	*1*	*2*	*3*	*4*
1. Event preview	.47	.50	-			
2. Task performance	8.58	3.05	.191*	-		
3. Team efficacy	3.57	.54	.135	.466**	-	
4. Openness of communication	4.48	.39	.193*	.105	.273**	-

*Note*: N = 119 teams. Event preview condition was coded as: 0 = no event preview, 1 = event preview.

*. Correlation is significant at the 0.05 level (2-tailed).

**. Correlation is significant at the 0.01 level (2-tailed).

Several independent *t*-tests were conducted using SPSS 26 to test Hypotheses 1 and 2. The event preview condition served as the between-subject independent variable in all analyses. For each dependent variable, the score means and standard deviations per condition are shown in Table 3.

**Table 3:** Dependent variable score means and standard deviations per condition.

Variable	Non-event preview		Event preview	
	*M*	*SD*	*M*	*SD*
Task performance	8.03	3.07	9.20	2.93
Team efficacy	3.50	.46	3.65	.61
Openness of communication	4.41	.37	4.56	.05

*Note*: N = 119 teams of which 63 non-event preview and 56 event preview.

### Hypothesis 1

Hypothesis 1 proposed that conducting an event preview would cause teams to score higher on task performance when compared to not conducting an event preview. We expected that these teams would answer more questions correctly than teams that did not conduct an event preview. This hypothesis was supported, as event preview teams scored significantly higher on task performance (*M* = 9.20, *SD* = 2.93) than non-event preview teams (*M* = 8.03, *SD* = 3.07), *t*(117) = -2.11, *p* = .037. We computed effect sizes according to Cohen’s d, which was 0.39.

### Hypothesis 2

Hypothesis 2 predicted that conducting an event preview would cause teams to report higher levels of openness of communication when compared to non-event preview teams. The analysis proved that teams that conducted an event preview indeed scored significantly higher on this measure, *t*(117) = -2.13, *p* = .036. Means of the event preview teams versus those of the non-event preview teams were 4.56 (*SD* = 0.05) and 4.41 (*SD* = 0.37) respectively. The effect size (*d*) was 0.57. Thus, Hypothesis 2 is supported.

### Hypothesis 3

Hypothesis 3 predicted that the positive effect of conducting an event preview on team efficacy would be mediated by the openness of communication. To test the indirect effect, we performed a mediation analysis using the process macro in SPSS 26. The unstandardized indirect effects and 95% confidence intervals were computed for each of the 5,000 bootstrapped samples [42]. The results did not indicate a significant effect of conducting an event preview on team efficacy, *R*^*2*^ = .14, *b* = 0.14, *SE* = 0.10, *t*(117) = 1.475, *p* > .05. However, in support of Hypothesis 3, the results indicate that the indirect effect is significant (*b* = 0.053, *SE* = 0.03; 95% CI [0.00, 0.12]). As expected, conducting an event preview positively affects a team’s openness of communication (*b* = 0.15, *SE* = 0.07; 95% CI [0.01, 0.29]). Additionally, the results show a significant effect of openness of communication on team efficacy (*b* = 0.35, *SE* = 0.13; 95% CI [0.11, 0.60]). Thus, Hypothesis 3 is supported.

## Discussion

The goal of the present study was to experimentally test the effectiveness of a new PM practice, the event preview. The results of our study indicated that teams conducting event previews were more effective–compared to teams who did not conduct an event preview–in terms of performance and openness of communication. Additionally, we found support for our assumption that the openness of communication mediates the positive effect of conducting an event preview on team efficacy [35, 36]. In summary, the results indicate that conducting an event preview can positively affect some important team-related outcomes. When an event preview is conducted, teams seem to be more productive, as they are able to provide more correct answers. Additionally, the perception of communication seemed to improve when teams conduct an event preview. This may cause individuals to communicate more openly, which may eventually improve their perceived team efficacy. The results suggest that an event preview is an effective practice that can be implemented before an event or project to achieve certain outcomes and improve overall performance. This indicates that using the strategy of mentally simulating an event may indeed enhance teamwork [10, 26]. Our results present the first impression of the event preview and the underlying psychological processes, which are based on CLT and the strategy of mental contrasting [10, 17, 20]. However, more research on the event preview process is needed to verify and confirm these suggestions.

### Theoretical and practical implications

The findings implicate that an event preview is a successful and easy intervention that may improve the effectiveness of the team in which it is conducted. By systematically discussing a fixed set of five questions, the upcoming event or project is examined within the team and a mental simulation is constructed. As our findings suggest that this simple and brief intervention may improve the performance of a team, it could be an interesting practice to implement in an organization. Organizations may implement the event preview when employees engage in a project together; for example, supervisors within the organization can provide these five questions and stimulate the team to discuss them together. Consequently, communication can be improved and possible issues are considered. By conducting a brief event preview, team members construct a mental simulation and make sure that they are on the same page, which may improve further communication [35, 36]. The intervention itself is inexpensive and easy to implement before the start of an event or project.

The event preview is a simple practice that may stimulate team members to reflect on upcoming events, which could foster their learning and improve performances. We argue that this innovative practice stimulates continuous conversation cycles in the organizations. This is in line with several calls in literature that argue for a new PM approach. This new approach is needed as scholars and practitioners argue that the traditional PM system no longer accommodates the daily workflow and activities of today’s organizations. Especially the annual progress reviews seem to demotivate employees and are not helpful in improving employees’ performance [2, 6, 7, 12]. Instead, researchers and practitioners advocate for an approach that stimulates monitoring and feedback [1, 2], as this would create more learning opportunities. Since the event preview meets the criticisms of traditional learning-oriented practices–i.e., it focuses on the future and not on the past, unlike other practices such as feedback and the AAR [1, 15]–it can be an addition to traditional PM practices. Incorporating event previews in the PM cycle and stimulating the use thereof when new projects are started, could possibly help employees to use new projects as learning opportunities. This can, in turn, be beneficial for employees’ and organizations’ agility and flexibility. We thus argue that this practice proposes a solution to the increasingly criticized traditional PM [1]. However, more research on this process is needed to confirm this claim. Additionally, further research on the effectiveness of an event preview in different organizational structures may also be useful.

### Limitations and Suggestions for Future Research

There are some limitations of the event preview and the present study that are noteworthy, as they may be useful for future research. First, within the event preview, the idea of mentally simulating the event is central. However, we do not know whether and to which extent it is possible to envision or simulate extremely uncertain and ambiguous situations. Whenever the project presented is entirely unknown, it might be difficult to imagine the accompanying obstacles or success factors that may occur. Future research should explore this question. Furthermore, unlike the PreMortem, an event preview encompasses interaction between team members. This requires a certain level of cooperation. Teams lacking a healthy team dynamic might find it more difficult to get ‘on the same page’ during an event preview. Finally, the event preview does not contain a post-project component to facilitate reflection. Reflection has been identified as an effective step in the learning process [43]. To tackle this limitation, the event preview can be combined with an AAR.

Secondly, teams were artificially composed by the voluntary participants of the experiment; thus, the event preview has not been proven useful in a real-life organizational context. Therefore, future research on the event preview with a larger sample and in a real-life context would be informative. Another limitation of the artificially composed sample may be the different degrees of familiarity within the teams. Participants independently assembled the teams, which means that teams may vary in their degree of familiarity. However, it is likely that most teams had a previous relationship with each other and had worked together in the past. This high degree of familiarity may have affected our results. A real-life study in an organizational context would resolve this limitation. Future research could also consider this limitation by focusing on the possible impact of familiarity within the team as a moderator.

The limitations of the conducted experiment are also worth noting. Participants were asked to discuss the five questions of the event preview before completing the puzzles. Although we attempted to capture how they executed the manipulation based on the team notes provided to us, future research could assure that the event preview is conducted appropriately by having a researcher observe the participants while the event preview is conducted. Furthermore, this experiment only tested the event preview when it was conducted in a team context. However, the event preview reflects on how individuals will tackle an event or activity that will take place in the near future. It can be executed by individuals who cooperate on a certain project but may also be useful when conducted by an individual in consultation. In this case, we only examined outcomes related to the version conducted in a team context. However, future research that focuses on the effects of the event preview when conducted by an individual and a supervisor may be interesting.

Lastly, we suggest that it may be interesting to explore the cumulative effect of implementing both an AAR and an event preview in a team context. The AAR has proven to be an effective practice with the aim of improving performance [9, 44]. Our research suggests that an event preview may have the same effect. Both practices are costless, simple, and brief interventions that can be easily implemented in an organization. If there is indeed a cumulative effect of both practices, this could be an important finding for companies looking to improve their PM.

### Conclusion

Despite the aforementioned limitations of this experiment, our study presents event preview as an innovative addition to traditional PM practices, as it solves for the current criticism on the topic. Namely, conducting an event preview can facilitate learning within the organization, as projects are closely monitored, planned, and seen as learning opportunities. Event previews stimulate team members to focus on the possibilities in the future rather than on mistakes in the past. Additionally, this study suggests that it is effective at enhancing several important team-related outcomes, such as team performance and the openness of communication. However, the effect of conducting event previews on other crucial performance-related outcomes remains unclear. More research is needed to make conclusive statements, but this study could serve as a stimulant for practitioners and researchers to further evaluate and assess the event preview practice and its effect on individual, team-related, and performance-related outcomes.
